# A Multilevel Model of Older Adults’ Appropriation of ICT and Acquisition of Digital Literacy

**DOI:** 10.3390/ijerph192315714

**Published:** 2022-11-25

**Authors:** Eija Kärnä, Lotta Aavikko, Rebekka Rohner, Vera Gallistl, Kaisa Pihlainen, Claudia Müller, Anja Ehlers, Roberta Bevilacqua, Stefano Strano, Elvira Maranesi, Katerina Cerna, Lisa Hengl, Franz Kolland, Franz Waldenberger, Gerd Naegele, Sieun Park, Moritz Hess, Verena Reuter, Susanne Frewer-Graumann, Kristiina Korjonen-Kuusipuro

**Affiliations:** 1 School of Educational Sciences and Psychology, University of Eastern Finland, 80101 Joensuu, Finland; 2 Division of Gerontology and Health Research, Karl Landsteiner University of Health Sciences, 3500 Krems an der Donau, Austria; 3 Information Systems, esp. IT for the Ageing Society, University of Siegen, 57072 Siegen, Germany; 4 Research Association for Gerontology (FfG)/Institute for Gerontology, Technical University Dortmund, 44339 Dortmund, Germany; 5 Scientific Direction, IRCCS INRCA, 60124 Ancona, Italy; 6 Department of Applied IT, MDI Division, Gothenburg University, 413 14 Gothenburg, Sweden; 7 German Institute for Japanese Studies, Tokyo 102-0094, Japan; 8 Hochschule Niederrhein, University of Applied Sciences, 47805 Krefeld, Germany; 9 Division of Health and Social Affairs, FOM University of Applied Sciences, 48155 Münster, Germany; 10 Juvenia-Youth Research and Development Centre, South-Eastern Finland University of Applied Sciences, 50101 Mikkeli, Finland

**Keywords:** older adults, digital literacy, digital skills training, multilevel model

## Abstract

Digital literacy refers to a set of competencies related to the skilled use of computers and information technology. Low digital skills can be a barrier for older adults’ full participation in a digital society, and COVID-19 has increased this risk of social exclusion. Older adults’ digital inclusion is a complex process that consists of the interplay of structural and individual factors. The ACCESS project unwrapped the complexity of the process and developed an innovative, multilevel model that illustrates how societal, institutional, material and pedagogical aspects shape adults’ appropriation of digital literacy. A holistic model describes factors contributing to older adults’ digital literacy, acknowledging sociocultural contexts, environments, learning settings and instruction practices for learning digital literacy. Instead of seeing older adults’ reasons for learning digital skills purely as individual choice, this model recognizes the interpersonal, institutional and societal aspects that implicitly or explicitly influence older adults’ acquisition of digital literacy. The results offer a tool for stakeholders, the research community, companies, designers and other relevant stakeholders to consider digital skills and the given support. It demands diverse communication between different stakeholders about the things that should be discussed when organizing digital support in digitalized societies.

## 1. Introduction

Digitalization is one of the defining characteristics of the contemporary era [1]. Consequently, the ICT revolution has driven global development in an unprecedented way for last two decades, and technological progress has brought unexpected growth in ICT access and connectivity to billions of people around the world. According to International Telecommunication Union (ITU) (2020) [2], in the middle of 2020, there were an estimated 105 mobile-cellular subscriptions per 100 inhabitants and at the end of 2019, over half of the world population was using the Internet. It has been estimated that ICTs will play an even more significant role in the future as an accelerator of economic growth and development and an important driver of progress towards the 17 Sustainable Development Goals (ITU, 2020) [2].

Besides digitalization, aging of the population is another megatrend in the world. According to the World Health Organization (2018) [3], the world’s population is aging quicker than ever before, and the number of people 60 years of age and over will increase from 12% to 22% of the global population by 2050. At the same time, it is suggested that ageism is increasing, thus causing adverse consequences for older people’s social inclusion. Ageism means that age is used to categorize and divide people in ways that lead to harm, disadvantage and injustice, and decrease of solidarity across generations (WHO 2021) [4].

At best, digitalization and the increased access and use of digital technology improve people’s quality of life, for example by facilitating access to health care services and enhancing social connectivity in general [5]. In addition, frequent connection to the Internet can promote mental and physical health, especially for older adults [6,7,8]. However, for decades, scholars and policymakers have expressed concern for groups of the population, e.g., older adults, facing uneven access to digital technology, e.g., Internet [2,9,10] and social inequities that influence how information is used, a phenomenon known as the digital divide [11,12].

In general, digital divide refers to the gap between those who do and those who do not have access to new forms of information technology [13,14,15]. At the social level, digital divide refers to the gap between the majority and minority groups including low-income residents, ethnic minorities, rural communities, elderly, the disabled, and women [8]. As Fang et al. [16] emphasize, more attention must be paid to intersectionality: different aspects of social inequality intensify each other and thereby impede access to digital technology. However, while early digital divide research looked at access, more recent research has focused on the capabilities and skills to use digital technology, i.e., digital literacy [12] and on the use of media across populations [11,17].

### Digital Literacy and Older Adults

Low information and communications technology (ICT) skills seem to remain a barrier to meaningful participation in a digital society, particularly for the older population [2]. In addition, COVID-19 has made the range of digitalization effects on older adults—from new opportunities for participation to new risks of social exclusion—more than clear [18,19,20,21]. This in particular applies to the education systems and possibilities for older people to participate in it [22,23], highlighting the importance of exploring how older people acquire digital literacy to ensure their digital inclusion.

According to Tsatsou [24], older adults’ digital inclusion is a complex process that consists of the interplay between structural/external and individual/internal factors and in which ageing plays a context- and life-experience–driven role. Consequently, it is necessary to consider dynamics among sociocultural and psychological/individual-level parameters in relation to older adults’ digital inclusion. This article explores these many parameters that influence older adults’ digital literacy. The leading question is: What parameters support or hinder older adults’ appropriation of ICT and the acquisition of digital literacy?

Digital literacy has been defined in many ways [25]. Generally, the concept has been referred to as a set of competencies related to the skilled use of computers and information technology [26]. Researchers [26,27] have suggested more elaborate definitions and have called for a reconsideration of digital literacy. For example, Calvani et al. [27] have suggested that digital literacy, which they also refer to as digital competence, consists of abilities ‘to explore and face new technological situations in a flexible way, to analyze, select and critically evaluate data and information, to exploit technological potentials in order to represent and solve problems and build shared and collaborative knowledge, while fostering awareness of one’s own personal responsibilities and the respect of reciprocal rights/obligations’.

Recent discourses on digital competencies have brought up various models. For example, in the eight German reports on ageing [28], the concept ‘digital sovereignty’ differentiates between three levels of digital competencies, which are helpful for the development of a wider understanding of appropriation of ICT and gaining digital literacy. The first level includes knowledge to operate and use digital media (use competence). The second level contains knowledge to adapt digital media to individual needs and to use media in creative ways (adaptation/design competence), and the third level (orientation competence) includes knowledge that people need to orientate themselves in the media landscape by being able to reflect on and evaluate digital possibilities [28].

The research indicates that digital literacy programs can empower older persons, foster social participation, and increase older adults’ autonomy. In addition, independence and that tailored peer- or intergenerational training initiatives targeted at older persons have proven to be effective in enhancing their digital literacy [29,30,31]. Martinez Alcala et al. [32] have introduced several key principles for the older adults’ digital literacy training. First, knowledge that is provided to older adults must be useful to learn and it has to respond to the older adults’ personal social needs. Second, training aiming at the improvement of older adults’ digital literacy should be cooperative and collaborative. This means that the instruction should include teamwork and interaction to achieve more proactive learning. Third, the training should also foster social inclusion by providing knowledge on possibilities to expand communication through the web with their friends and relatives. Fourth, the training should promote older adults’ autonomy so that they could be the protagonists of their own learning. Therefore, the content of the training should be designed considering the older adults’ learning styles, interests and expectations of the senescent individual.

There are also studies that have introduced general instructional design and contextual considerations for delivering technology-based instruction to older adults [33,34,35]. For example, Wolfson et al. [35] have suggested that technology-based instruction for older adults should (1) be highly structured, (2) provide adequate feedback and adaptive guidance for older adults, (3) inform older adults on cognitive and metacognitive strategies they could facilitate learning, (4) utilize the principles of theories on individuals’ mental-processing capacity, and (5) include a user interface that is simple and consistent throughout the training. In addition, Wolfson et al. [35] have further emphasized that efforts should be taken to create contextual conditions that improve self-efficacy and training motivation among older adults and that further research should be conducted to determine if there is a need for age-specific instructional formats. Consequently, more research is needed to enhance older adults’ digital literacy and to reduce the digital skills’ gaps [36,37].

In this article, digital literacy is understood as a broad concept including premises from social theory of digital literacy and elements of digital sovereignty concept. The findings of recent research on older adults’ digital literacy indicate that digital technology uptake and appropriation are characterized by tensions such as images of ageing, forms of technology acceptance, attitudes and stakeholder interests [38]. Consequently, more research is needed to enhance older adults’ digital literacy and to reduce the digital skills gaps [37]. The ACCESS project tackled this issue and developed a multilevel model to illustrate how societal, institutional, material and pedagogical aspects shape older adults’ appropriation of ICT and the adoption of digital literacy.

## 2. Materials and Methods

### Contextualising the Model: ACCESS

The goal of the ACCESS project was to tackle multi-level challenges related to older adults’ digital literacy [24] by exploring, implementing and evaluating different modes of socially embedded learning opportunities for older adults to gain experiences and sustainable knowledge and skills regarding modern technology.

Furthermore, the project aimed to develop recommendations and a model for the set-up of a new learning culture around the uptake of older adults’ digital literacy. To reach the goals, the project explored different learning settings, such as formal courses to learn to use the internet, or non-formal settings such as peers supporting older adults to learn digital literacy skills. In addition, ICT learning practices of older adults were observed in the context of a long-term participatory design (PD) project, which aimed for the co-production of learning tools and a training concept for learning providers. The project combined expertise from five participating countries and from different disciplines, i.e., gerontology, socio-informatics, sociology, special education, usability engineering and cultural sciences.

The data used for the construction of the model was implemented in three phases. First, country-specific framework conditions concerning recent developments, policies and opportunities that supported or hindered older adults’ digital literacy were identified by reviewing national reports, policies and information from existing projects, initiatives, services, etc. In the second phase, the consortium partners reviewed current studies examining the interaction between older people and technology to gain a holistic picture of current state-of-the-art debates and existing gaps in the literature regarding older adults’ learning through and with technologies. In the third phase, data used for the construction of the model were collected during country-specific studies (see Table 1). All partners obtained an approval or the approval was waived according to the guidelines of the ethical committees of their organizations.

The focus of the Austrian research was on the role of technological artefacts in older adults’ engagement with new technologies. The data were collected on the one hand by interviewing older non-users of digital technologies in rural and urban areas of Austria, and on the other hand by using participatory observations, visual diaries and semi-structured interviews with trainers and participants of digital skills training courses.

The Finnish research focused on older adults’ acquisition of digital skills in formal and non-formal learning environments. The first data set was collected in spring 2019 by using a questionnaire for teachers and older adults participating in digital skills training courses in the adult education centres. Another data set was collected in spring 2020 by interviewing tutors and trainees in non-governmental organisations providing digital training for people of a similar age. The data were analyzed by using qualitative content analysis and descriptive statistical analysis.

In Germany, (A) conducted a quantitative study on digital technology courses for older adults by using questionnaires regarding reasons for course participation, self-rated ICT skills, use of digital applications, attitudes towards digital technology, and sociodemographic data. Course participants were surveyed in three waves from August 2019 until January 2021. Data analysis was carried out with descriptive statistics. In addition, from May to July 2021, qualitative interviews were conducted with experts regarding older adults’ digital skills training. Their answers were analyzed with qualitative content analysis according to Mayring [39].

Germany (B) conducted interviews and participant observations (partly with video recordings) with older adults in the context of a long-term participatory design project, with a focus on ICT learning support measures that help enable them to become qualified co-designers. The project aimed for the co-development of a toolbox and a training concept for supporting older adults in their appropriation of internet applications and assistive tools for domestic contexts. The researchers worked with 21 older adults in regular workshops over more than two years.

The research conducted in Italy focused on pilot training via ICT online course sessions, conducted in November 2020. The data were collected by a questionnaire delivered to the participants at the beginning and at the end of the pilot to quantify the improvements achieved, following the implementation of the training. Additional information was acquired by semi-structured interviews with experts in teaching digital skills to older adults. The description of the methods and results of the Italian pilot study are available in [40].

The research in Japan focused on scrutinizing the policies, financing and use of ICT-based solutions in Japan offered by local non-governmental organizations (NGOs) to support and prolong independent living of older people in their accustomed local surroundings. The data were collected via expert interviews with policymakers, social entrepreneurs, and Japanese academic demography experts, as well as via a survey with participants and teachers of ICT courses for the elderly offered by two NGOs in Tokyo.

The findings from the three stages of the data collection and analysis were shared and discussed repeatedly in the project meetings, and subsequent findings on contextual factors that promote or hinder digital literacy among older adults started to emerge. As a result, a holistic model describing factors contributing to older adults’ digital literacy from different angles, acknowledging (1) sociocultural contexts, (2) environments, and (3) learning settings and instruction practices for learning digital literacy, was created.

## 3. Results

**Figure 1:**
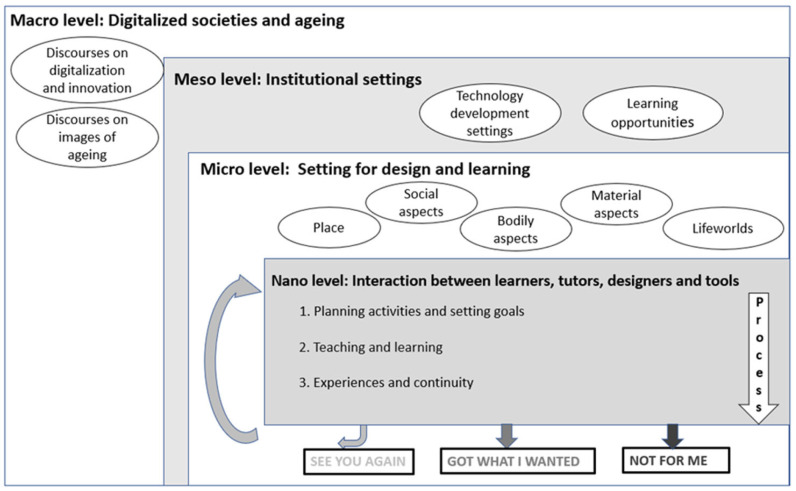
A multilevel model of parameters influencing older adults’ appropriation of ICT and acquisition of digital literacy.

Older adults’ appropriation of ICT and acquisition of digital literacy involve multi-level processes. The model presented below illustrates what parameters at the macro-, meso-, micro- and nano-levels need to be considered for gaining a holistic understanding of the complexity of parameters related to older adults’ digital literacy (Figure 1). In particular, for the macro level, such as the digitalized societies and ageing, discourses on digitalization, innovation and images of ageing should be taken into account. Furthermore, technology development settings and learning opportunities are the two aspects for the meso level. Instead, the micro level is characterized by place, lifeworlds, social, bodily and material aspects. Finally, for the nano level, that is, the interaction between learners, tutors, designers and tolls, three parameters need to be considered: planning activities and setting goals, teaching and learning, experiences and continuity.

### 3.1. Macro Level

Discourses on the design and use of digital technologies as well as images of ageing need to be considered, as they shape the way in which older adults’ needs for the adoption of digital literacy skills are perceived and supported in society [41]. Currently, discourses on innovation, technology and ageing typically focus on the facilitative and money-saving effects of information technologies on older adults’ lives, like enabling independent living [42], and increasing individual well-being [43]. However, more attention must be paid to socio-structural aspects at the macro level [16] and to transitions in a person’s life course, e.g., from paid work to retirement and the related loss of social roles, becoming a family caregiver, widowhood, etc. [44].

Furthermore, the current ageing and innovation discourse seems to be based on a negative view of ageing [45], which can contribute to problems with acceptance of these technologies and older adults’ willingness to use ICT [46]. The data from different countries in the ACCESS project confirm the abovementioned findings. On the one hand, some older adults expressed feelings of incompetence and reluctance to learn to use digital technology. Two interviewed experts put the possible effects of negative images of ageing as follows:

*“Doubt about one’s own ability to learn is a big issue, I think. So: ‘I’m not up to it anymore!’, and so on, ‘I’m too old for that.’”*

*(Germany, expert* *interview)*

*“[…] because they themselves think: ‘I won’t learn this anymore, I can’t do it.’ That they actually deny themselves the competence […]”*

*(Austria, expert* *interview)*

On the other hand, there were also older adults who already had the skills to use digital services and devices, and who were eager to learn more and to improve their digital literacy skills. Therefore, it is crucial that the support given to older adults to learn digital literacy skills and the appropriation of new technology is carefully planned and implemented in a way that considers older adults’ needs and their reasons for seeking guidance [47].

### 3.2. Meso Level

The institutional settings of technology development and learning opportunities also influence older adults’ participation in the design and use of digital technologies in everyday use. The advantages of older adults’ participation in PD have been acknowledged [48], but a considerable number of studies raise concerns about if and how involvement can make a difference in actual design practices.

To achieve acceptable technologies for older adults, the understanding of how they can be involved successfully in technology design and how to support the adoption of digital literacy skills during long-term participatory design processes are necessary [49]. In addition, there is increasing demand for sustainable implementation of the results obtained with co-researchers in long-term co-design projects, e.g., in the form of work in living labs. This includes technological results, but also new practices that the co-researchers have learnt in dealing with ICT [50].

Institutional opportunities for older adults to obtain support for the acquisition of digital literacy is related to national policies on digitalization. As part of the ACCESS project, a stocktaking of relevant policy framework conditions in the participating countries and national measures related to the topic ‘ageing and digitalization’ was conducted. It illustrated somewhat considerable differences in the way national policies consider and emphasize the subject.

According to UNECE [37], a large majority of countries have developed national strategies for digitalization that address the need for broadband infrastructure (including broadband access) and access to technological devices, as well as encouraging lifelong learning. However, few strategies explicitly address the specific needs of older people in relation to adopting digital literacy, and the benefits they may reap because of becoming more digitally literate. For example, supporting the internet use of older adults in Austria remains a marginal topic in policy papers, and lifelong learning is outlined as the most important one-size-fits-all solution. This is somewhat problematic, as Gallistl and colleagues [51] show that only a very small percentage of older adults can be reached by lifelong learning.

In Finland, a national operating model for the adoption of digital literacy skills describes how the availability of digital support can be organized [52]. The model is built by many actors and in strong cooperation with different stakeholders. Thus, it is flexible, considers the changes in digital formats and consequently the needs for the digital support, and is parallel with the development of digital society.

Although Japan has one of the highest internet penetration rates in the OECD, the digital divide between the younger and older generations (55–74 years) is relatively high [53]. In addition, there is a massive gap between the availability of off-the-shelf technology that potentially could support older adults to support their health management, mobility and social participation, and the actual status of their implementation is at an infancy stage. So far, policies have mainly focused on research and development; however, a recent initiative ‘Assistant for Digital Activities of Seniors’ aims at training assistants of local NGOs and personnel in retail shops selling ICT equipment to assist older adults in local communities.

There is also a connection between health and digital literacy. As health services are increasingly provided online, both health and digital literacy skills are simultaneously needed. Health technology interventions, health literacy training and services are often used to facilitate older adult health condition management [54]. However, they are not always designed to include a necessary learning phase before the introduction of a new technology, which could, in turn, strengthen the older adults’ digital literacy skills and the use of eHealth services. Therefore, the availability of digital skills learning opportunities is crucial for the development of older adults’ health and digital literacy skills [40].

### 3.3. Micro Level

The use of digital technologies forms new social practices and therefore influences everyday life in various ways. Although those practices are shaped by the design and possible applications of technology, they are not entirely determined by it [55]. Older adults’ use of digital technology also depends on their competencies and interests [55] as well as the opportunities for learning and updating their digital literacy skills. Therefore, the practices of using new technologies are embedded not only in discourses about ageing and institutional settings, but also on the instruction methods used to support the adoption of digital literacy skills. The important factors influencing the adoption of digital literacy skills are place, social, bodily and material aspects and the lifeworlds of older adults. With the term ‘lifeworld’, we refer to Alfred Schütz’s definition of the way we perceive and understand the world that surrounds us, based on our cultural experiences and knowledge [56].

#### 3.3.1. Place

Places are not ‘neutral’–meanings are assigned to different physical spaces, as different behaviors and practices could be (and are expected to be) enacted in them. Hence, learning how to use a computer at a local library will be enabled in a different way from learning how to use a smartphone at home. Older adults are surrounded by digital technology in their everyday lives. Therefore, their actual encounters with digital technology and the internet happens outside of course settings and in many different places, such as while observing younger people using digital technology in public spaces or when using an ATM [57]. Hence, the learning process is not restricted to the course setting, but it happens in various places [58].

#### 3.3.2. Societal Aspect

Older adults are a heterogenous group regarding their socio-economic background, living conditions, learning experiences and resources, which influence their access to and usage of the internet [51]. A study of older non-users in Austria [51] showed the heterogeneity of older non-users by differentiating between four different clusters. The first group, ‘younger non-users’, were significantly younger and in better health than in the other clusters. The second group was labelled ‘male non-users’, including solely males who mostly had greater financial resources. ‘Urban non-users’ had the highest percentage of adults living in urban environments and the highest educational backgrounds. Finally, ‘non-users with health limitations’ were the oldest and had the highest degree of health limitations.

While younger and urban non-users were likely to be reached by educational programmes, non-users with health limitations and male non-users showed hardly any participation in learning activities [51]. Therefore, learning programmes need to consider the heterogeneity of older users and especially of older non-users to go beyond one-size-fits-all solutions. However, in the context of non-users, social contacts are mostly referred to as a barrier to appropriation, because of a lack of support or avoidance of using the internet directly through proxy usage [57].

#### 3.3.3. Bodily Aspect

Research has highlighted that older adults face health-related barriers when learning digital technologies, arguing that learning how to use the internet is more challenging for those with poor eyesight, shaky hands or (mild) cognitive impairment, for example [59]. Furthermore, studies show that engaging with digital technology also contributed to particular images and experiences of age and ageing. Not being able to use (or learn) digital technologies in the way they are intended often meant that older adults experienced themselves as old, frail or excluded from society [57].

#### 3.3.4. Material Aspect

Digital devices were regarded as highly complex by both trainers and participants in digital literacy training. The complexity lies in their diverse operating systems and functions, but also that those functions are continuously changing, e.g., through updates. The complexity needs to be handled by the trainers, which they mostly do by being flexible and open to questions and problems that the digital devices bring up during the courses. However, this flexibility is perceived by many older adults as something that leads to unstructured and chaotic learning situations, and some find it boring [58,60].

Next to digital devices, there were also many other materials that were important for digital learning process in later life, such as notepads, pencils and step-by-step guidelines. Through those materials, digital technologies were perceived as if they needed to be studied: One must study the correct vocabulary and combinations of steps, write it down and practise it. This discourse is materialised through different materials, which made participants feel like they were part of a learning situation [58]. This highlights that material aspects need to be considered in a broader perspective, namely as a view of the ‘digital ecologies’ that surround older people in their daily lives [61], which might include different non-digital and digital materials with different operating systems.

#### 3.3.5. Lifeworlds of Designers/Instructors/Learners 

Older adults’ perceptions of their own ageing, well-being, learning and attitudes towards digital literacy affect the learning of digital skills. Especially if they see themselves as fragile and unable to learn new things, tutoring in digital skills from a peer who understands these influences can be especially vital and effective. To see actual benefits from digital skills in one’s personal life is significant in order to motivate older adults to learn more about the use of digital devices [47]. To organize such situations, the organizers would benefit from information beforehand about the interests, needs and special requirements of the tutored group [62].

### 3.4. Nano Level

The nano level also has an impact on older adult’s learning of digital literacy and their appropriation of ICT. It includes training activities and methods that either support or hinder the learning and appropriation processes. In addition, activities at the nano level contribute to older adults’ experiences of digital technology and learning of digital literacy skills. At best, older adults feel that training was beneficial for them, which encourages them to learn new digital literacy skills in the future [47]. According to the (name deleted for anonymity) project results, the training process typically has three interrelated, partly overlapping phases.

#### 3.4.1. Planning Activities and Setting Goals

When the training begins, it is crucial that the trainer collects some information about the older adults participating, particularly about their knowledge and skills in relation to the topic to be learnt [62]. Setting goals is easier if the participants can conceptualize what they want to learn. Thus, if the trainer is not able to retrieve this information in advance, as might be the case in more open-ended learning opportunities, it is necessary to have a range of different resources (e.g., plan to use different training methods or devices) at hand, which can be flexibly fitted to older adults’ learning needs. As one of the peer tutors said in an interview, their goal is often times to get the tutee to see “that this [technology] ain’t so very bad” (Finland, male peer tutor).

#### 3.4.2. Applying Training Styles, Collaborating, Participating, Supporting, Interacting with Devices and Tools

Training activities include the application of different training styles that are implicitly or explicitly based on different learning theories. The general principles regarding older adults’ learning of digital literacy skills, such as an emphasis on cooperative and collaborative learning and the promotion of older adults’ autonomy [35,63], provide overall guidelines for training. In addition, active participation of older adults is important in the training. “There we’re closer to more equal than with a youngster whom goes on so quickly [with tutoring]” (Finland, male peer tutor). Sometimes, the participating older adults may know more than a trainer which, at best, can increase the collaborative learning of all participants [47]. In addition, the application of three main learning theories, namely behaviorism, cognitivism and social constructionism, is an efficient way to support older adults to achieve digital competencies [64,65].

#### 3.4.3. The End of the Training Session, including Experiences and Continuity

It is important to acknowledge the experiences of older adults, as they help the trainers to further develop the content and methods of the training. As the interviews put it: “Yes it has been quite nice to tutor. And when you see, that it has been beneficial for someone. You can see that they have learnt to use [technology]” (Finland, female peer tutor). If the training does not meet the needs of the older adults, their experiences can be negative and foster their exclusion from future digital training opportunities [60]. Therefore, it is crucial to acknowledge each learner in the situations and ensure their experiences are heard.

## 4. Discussion

The model presented in this article sheds light on the various aspects at the macro, meso, micro, and nano level that support or restrict older adults’ digital literacy and appropriation of ICT. Instead of seeing older adults’ reasons for learning digital skills purely as individual choice, this model recognizes the interpersonal, institutional and societal aspects that implicitly or explicitly influence older adults’ acquisition of digital literacy. In addition, the model highlights cultural contexts, aspects of sustainability of learning practices, and learning offers. For example, discourses on digital innovations, business development and new business models must be closely linked to the consumer perspective of all citizens, including older adults.

When promoting learnability, it is also essential to address the specific learning needs and contexts of older adults. As Schirmer et al. [62] have argued, new technological innovation and new social practices in digitalized societies may be alienating, particularly for digitally illiterate older people. Thus, the tasks of digital skills trainers go beyond that of ‘regular’ teachers. At best, digital skills training considers the lifeworld of older adults by connecting content and pedagogics to the older adults’ needs, values and desires [62].

The model indicates that older adults’ experiences are crucial in understanding and supporting their digital literacy, as they are reflected in their reasons to enter, re-enter or withdraw from the digital skills learning situations [60]. In these multiple scenarios lies a question about how we fill the gap from ‘a person with some competence that can navigate in the digital landscape to serve their own needs’ to ‘a person that fully understands the datafication processes’. This viewpoint reflects the discourse on ‘digital sovereignty’ [28], a holistic concept of an autonomously acting individual that is fully capable of taking informed decisions in the digital world. This shift requires more research on design practices as well as on the digital skills of teachers and tutors. For instance, the results of the training conducted in Italy, which was related to the domains combining digital and health literacy (i.e., eHealth), confirm that older adults who participated in ICT courses increased their theoretical knowledge of the digital world, the skills being used, and technological awareness. This evidence is even stronger in the case of participants who possessed low levels of digital literacy prior to the training course and is much more marked in the area of eHealth literacy, demonstrating that an adequate learning model that is more standardised and tailored to the needs of older adults is significantly effective. An outcome with high potential suggests that there is currently a lack of suitable training formats targeting older adults and joining health and digital literacy [54].

To counteract the common misguided developments in technology research, the demand for sustainability of practice-oriented research projects is becoming louder [66]. This could be used as an opportunity to build stronger bridges between long-term, local research and co-design projects with the participation of NGOs, older participants and local companies. Then, learning could be conceived as multi-lateral learning between user groups, trainers and researchers as regional development approaches to ultimately contribute to futureproofing regions.

## 5. Conclusions

Fostering digital competences and literacy in older people has the potential to improve psychological wellbeing, health and inclusion of the older adults in the rapidly technological-changing society. Currently, there are frameworks and models that try to understand the digital learning needs of the older people, but not one of them is dominant. Moreover, there is the need to combine the above-mentioned frameworks with practical and proved strategies to be easily taken up in different settings and contexts, in order to counteract the older people’ exclusion from the digital world, globally.

In order to effectively address this gap, a comprehensive approach is required, building on innovative evidence and best practices, to raise awareness in citizens, communities, professionals, and health and social care providers, including also the ‘hard-to-reach’ people, often excluded by the digital world.

The ACCESS multilevel analysis reveals the complexities of the phenomenon, and helps in further developing research, policies and practices concerning ICT learning support of older adults. The model based on our interdisciplinary and multi-country study highlights the need to recognize older people’s digital learning as complex and socially embedded practices that differ in part from ICT learning and appropriation practices of younger cohorts. A particularly important factor is the need for low-threshold and long-term learning opportunities, either in the form of local formal or informal offerings that can elicit and address individual interests alongside age-friendly didactics. In addition, companies need to be pressured to make the learning-friendliness of their applications a priority, either legally or as a selling point.

There were some limitations to this study. First, the amount of data collected as well as the number of participating countries in the consortium were limited. Therefore, generalizations cannot be made. In addition, participants used different data collection methods in their country-specific studies and thus the comparison of the results was difficult. Furthermore, even though the model is multilevel, it is static. Therefore, future research should focus more on the dynamic processes of older adults’ adoption of digital literacy.

## Figures and Tables

**Table 1:** Participants, data collection and analysis methods.

Country	Participants	Data Collection Methods	Data Analysis
**Austria**	15 older non-users of digital technology (69–88 years); nine participants (61–81 years) and seven trainers of five ICT courses (23–77 years)	Interviews Visual diary Participant observation	Qualitative analysis
**Finland**	156 participants of ICT courses (65–89 years) 8 peer trainees (70–81 years)	Questionnaire Interviews Participant observation	Statistical analysis Qualitative analysis
**Germany (A)**	214 participants of ICT courses (51–89 years) 7 experts	Questionnaire Interviews	Statistical analysis Qualitative analysis
**Germany (B)**	17 participants of long-term participatory design sessions (65–69 years)	Interviews, participant observation, video recordings of participatory design sessions	Qualitative analysis
**Italy**	58 participants subdivided into three groups/ICT online courses (50–77 years)	Questionnaires Interviews	Statistical analysis
**Japan**	10 experts: policymakers, social entrepreneurs and demography scholars 19 participants and teachers of ICT courses (50–89 years)	Expert interviews Questionnaire	Qualitative analysis Descriptive statistical analysis

## Data Availability

Not applicable.
